# Early Bleeding Risk After DOAC Initiation With Concomitant Amiodarone in Older Adults With Atrial Fibrillation

**DOI:** 10.1111/jce.70440

**Published:** 2026-07-08

**Authors:** Isadora D. Guarino, Saman Nazarian

**Affiliations:** ^1^ Department of Internal Medicine Hospital of the University of Pennsylvania Philadelphia Pennsylvania USA; ^2^ Cardiac Electrophysiology Section, Division of Cardiovascular Medicine Hospital of the University of Pennsylvania Philadelphia Pennsylvania USA

**Keywords:** amiodarone, atrial fibrillation, bleeding, direct oral anticoagulants, drug–drug interaction

## Abstract

**Background:**

Amiodarone is frequently co‐prescribed with direct oral anticoagulants (DOACs) in atrial fibrillation (AF), but its impact on early bleeding risk after DOAC initiation in older adults remains uncertain. We evaluated short‐term safety outcomes associated with concomitant amiodarone use at the time of DOAC initiation.

**Methods:**

We performed a retrospective propensity score matched cohort study using the TriNetX US Research Network (2015 to 2025). Adults aged 75 years or older with nonvalvular AF newly initiating a DOAC were included. Exposure was defined as amiodarone prescription or administration within 30 days before or on the DOAC start date. Patients were matched 1:1 on demographics, comorbidities, and baseline cardiovascular medications. Follow‐up began 1 day after DOAC initiation and continued for 90 days. Primary outcomes were gastrointestinal (GI) bleeding and intracranial hemorrhage (ICH). Ischemic stroke was a secondary outcome.

**Results:**

After matching, 136,806 patients were included (68,403 per cohort), with all standardized mean differences < 0.1. Index‐date DOAC exposures were predominantly apixaban (82.7%) and rivaroxaban (16.5%). Concomitant amiodarone use was associated with higher early bleeding risk. GI bleeding occurred in 2.3% vs 1.9% (HR 1.30, 95% CI 1.20 to 1.40), and ICH occurred in 0.4% vs 0.3% (HR 1.28, 95% CI 1.07 to 1.52). There was no significant difference in ischemic stroke (1.3% vs 1.5%; HR 0.91, 95% CI 0.83 to 1.01).

**Conclusions:**

Among adults aged 75 years or older initiating DOAC therapy, concomitant amiodarone exposure was associated with a modest but significant increase in 90‐day GI bleeding and ICH without a detectable increase in ischemic stroke. These findings support careful medication review, dose selection, and early bleeding counseling when amiodarone is used with a DOAC in older adults.

**Abbreviations**
AF: atrial fibrillation
CI: confidence interval
DOAC: direct oral anticoagulant
GI: gastrointestinal
HR: hazard ratio
ICD‐10‐CM: International Classification of Diseases, Tenth Revision, Clinical Modification
ICH: intracranial hemorrhage
RR: risk ratio

1

Amiodarone is commonly co‐prescribed with direct oral anticoagulants (DOACs) in atrial fibrillation (AF), yet the safety of this combination during the early initiation period remains uncertain. Amiodarone inhibits P‐glycoprotein and CYP3A4, pathways that are central to DOAC absorption and clearance, and expert guidance recognizes the potential for clinically meaningful drug‐drug interactions when DOACs are used with inhibitors of these pathways [1, 2]. However, the magnitude of bleeding risk, particularly during the early period after DOAC initiation when patients may be most vulnerable, remains incompletely defined [3, 4, 5].

We conducted a retrospective, propensity score‐matched cohort study using the TriNetX US Research Network (January 2015 through January 2025). We identified adults aged 75 years or older with nonvalvular AF who newly initiated a DOAC (apixaban, rivaroxaban, dabigatran, or edoxaban). Patients with prosthetic heart valves or rheumatic mitral stenosis were excluded. To support a new user design, individuals were required to have at least one recorded health care encounter in the 365 days before DOAC initiation. Exposure was defined as a recorded amiodarone prescription or administration within 30 days before or on the DOAC initiation date; the referent cohort had no amiodarone exposure during this window. Follow‐up began 1 day after DOAC initiation and continued for 90 days, with censoring at outcome occurrence or last recorded encounter. Propensity score matching (1:1 nearest neighbor) was performed using age, sex, race, heart failure, ischemic heart disease, prior cerebrovascular disease, chronic kidney disease, diabetes, hypertension, and baseline cardiovascular medications, including beta blockers, diuretics, renin angiotensin system inhibitors, statins, antiplatelet agents, and digoxin.

The primary safety outcomes were gastrointestinal (GI) bleeding and intracranial hemorrhage (ICH). A key secondary outcome was ischemic stroke. Event risks and hazard ratios (HRs) were estimated using TriNetX time‐to‐event methods (Kaplan–Meier and Cox models). After matching, 136,806 patients were included (68,403 per cohort), and all standardized mean differences were < 0.1. At the index date, recorded DOAC exposures were predominantly apixaban (82.7%) and rivaroxaban (16.5%), whereas dabigatran (0.7%) and edoxaban (0.1%) exposures were uncommon. Amiodarone exposure at the time of DOAC initiation was associated with a higher risk of early bleeding: GI bleeding occurred in 2.3% vs 1.9% (HR 1.30, 95% CI 1.20–1.40), and ICH occurred in 0.4% vs 0.3% (HR 1.28, 95% CI 1.07–1.52), shown in Figure 1. There was no significant difference in ischemic stroke (1.3% vs 1.5%; HR 0.91, 95% CI 0.83–1.01; log rank *p* = 0.07).

**Figure 1:**
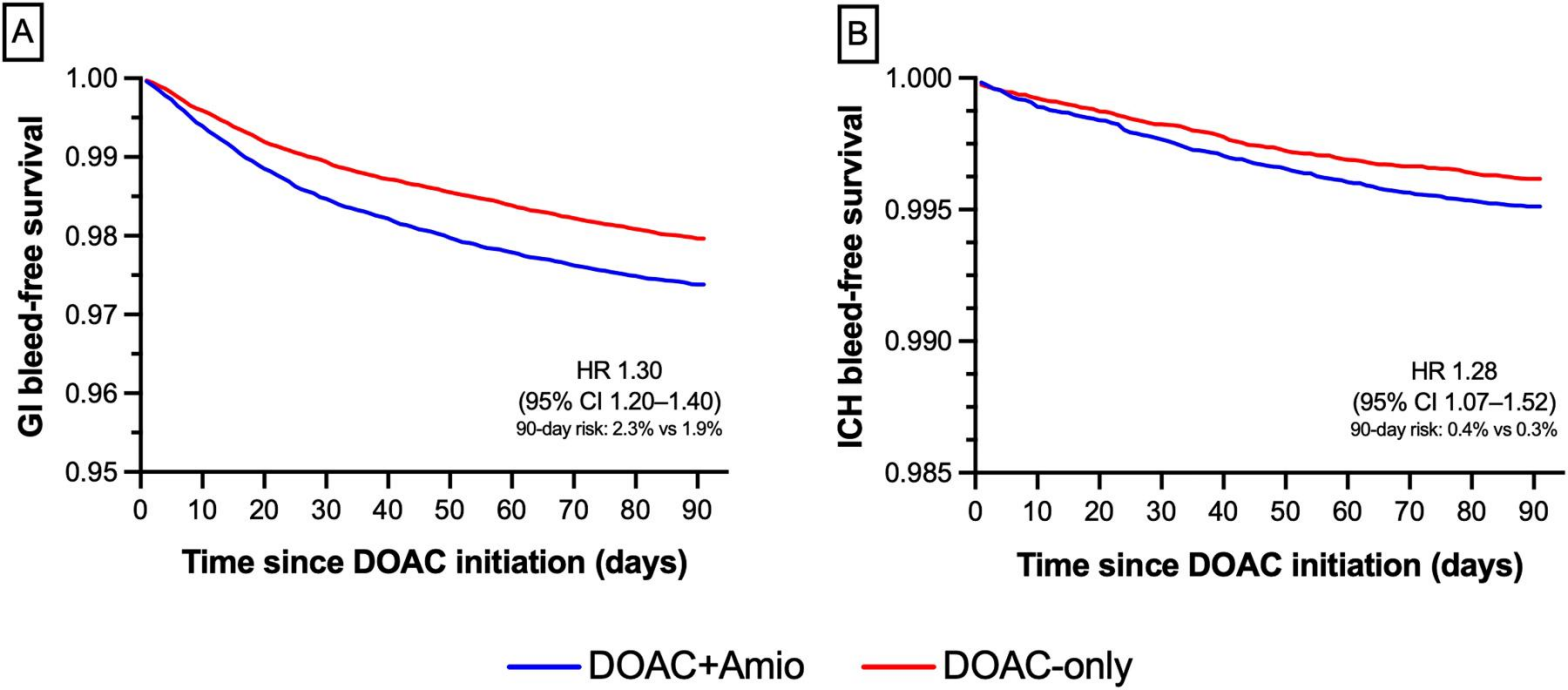
Kaplan–Meier curves for (A) gastrointestinal bleeding and (B) intracranial hemorrhage within 90 days following DOAC initiation in matched cohorts of adults aged ≥ 75 years with nonvalvular AF, comparing amiodarone exposure versus no amiodarone exposure in the same window.

In this large matched cohort of adults aged 75 years or older, concomitant amiodarone use around the time of DOAC initiation was associated with a modest but statistically significant increase in early GI bleeding and ICH, without a detectable increase in ischemic stroke over 90 days. These findings are consistent with prior observational studies showing higher bleeding‐related hospitalizations with concurrent amiodarone and factor Xa inhibitors [3, 4], and with a recent meta‐analysis demonstrating a reproducible association between amiodarone exposure and bleeding among DOAC‐treated patients across real‐world datasets [5]. Together with established guidance recognizing P‐glycoprotein and CYP3A4‐mediated DOAC interactions [1, 2], our results support careful medication review and early bleeding risk mitigation when initiating DOAC therapy in patients also receiving amiodarone.

Key limitations of our study include residual confounding by indication (patients receiving amiodarone may have greater arrhythmia burden, frailty, or acute illness), incomplete capture of DOAC dosing and adherence, and outcome misclassification inherent to code‐based EHR analyses. However, the prespecified early follow‐up window, biologic plausibility of an interaction, and the consistent increases across clinically important bleeding outcomes support a credible safety signal. Given the severity and high morbidity of ICH, even small absolute risk differences may be clinically meaningful in adults aged 75 years or older.

In conclusion, among adults aged 75 years or older initiating DOAC therapy, concomitant amiodarone exposure around initiation was associated with higher 90‐day rates of GI bleeding and ICH, without a detectable difference in ischemic stroke. These findings support careful medication review, dose selection, and patient counseling about early bleeding symptoms when amiodarone is prescribed with a DOAC. Further studies should evaluate whether risks vary by individual DOAC and dosing and identify practical strategies to reduce early bleeding in this high‐risk population.

## Conflicts of Interest

The authors declare no conflicts of interest.

## Data Availability

The data that support the findings of this study are available from the corresponding author upon reasonable request.
